# Small RNAs as important regulators for the hybrid vigour of super-hybrid rice

**DOI:** 10.1093/jxb/eru337

**Published:** 2014-08-16

**Authors:** Lei Zhang, Yonggang Peng, Xiaoli Wei, Yan Dai, Dawei Yuan, Yufei Lu, Yangyang Pan, Zhen Zhu

**Affiliations:** State Key Laboratory of Plant Genomics and National Centre for Plant Gene Research (Beijing), Institute of Genetics and Developmental Biology, Chinese Academy of Sciences, No. 1 West Beichen Road, Chaoyang District, Beijing 100101, PR China

**Keywords:** Auxin signalling pathway, differentially expressed gene, heterosis, QTL mapping, rice, small RNA, transcriptional profile.

## Abstract

Heterosis is an important biological phenomenon; however, the role of small RNA (sRNA) in heterosis of hybrid rice remains poorly described. Here, we performed sRNA profiling of F_1_ super-hybrid rice *LYP9* and its parents using high-throughput sequencing technology, and identified 355 distinct mature microRNAs and *trans*-acting small interfering RNAs, 69 of which were differentially expressed sRNAs (DES) between the hybrid and the mid-parental value. Among these, 34 DES were predicted to target 176 transcripts, of which 112 encoded 94 transcription factors. Further analysis showed that 67.6% of DES expression levels were negatively correlated with their target mRNAs either in flag leaves or panicles. The target genes of DES were significantly enriched in some important biological processes, including the auxin signalling pathway, in which existed a regulatory network mediated by DES and their targets, closely associated with plant growth and development. Overall, 20.8% of DES and their target genes were significantly enriched in quantitative trait loci of small intervals related to important rice agronomic traits including growth vigour, grain yield, and plant architecture, suggesting that the interaction between sRNAs and their targets contributes to the heterotic phenotypes of hybrid rice. Our findings revealed that sRNAs might play important roles in hybrid vigour of super-hybrid rice by regulating their target genes, especially in controlling the auxin signalling pathway. The above finding provides a novel insight into the molecular mechanism of heterosis.

## Summary

Differentially expressed miRNAs and ta-siRNAs between hybrid rice and its parents play important roles in hybrid vigour of super-hybrid rice by regulating their target genes controlling the auxin-mediated signalling pathway.

## Introduction

Heterosis, or hybrid vigour, is the superior performance of hybrids in biomass, resistance, and fertility compared with their parents (Shull, 1908; Bruce, 1910). Heterosis has been widely applied in the breeding of agricultural crops, and its underlying mechanism has been studied for over 100 years (Darwin, 1876). Hybrid rice is one of the most successful examples taking advantage of heterosis in crop breeding. Recent statistics showed that the planting area of hybrid rice accounts for more than 50% of the total rice area in China (Cheng *et al.*, 2007), further indicating that hybrid rice plays an important role in international food security by increasing grain yield. Therefore, there is an urgent need to explore the molecular mechanisms associated with heterosis, to provide a foundation for further utilization of heterosis. Rice has a relatively small genome size, high-quality sequences, and has colinearity with other cereal species in the Poaceae family (Gale and Devos, 1998; International Rice Genome Sequencing, 2005). Importantly, there are also many databases and bioinformatic tools for rice, including rice genome annotation and gene expression, pathway, mutant library, and phenotype information data (Zhang *et al.*, 2013). Thus, hybrid rice is an optimal model plant for genome-wide studies of heterosis.

The mechanism of heterosis has been studied extensively and there are three classic hypotheses to explain heterosis, with each explaining the genetic mechanism to some extent (Goff and Zhang, 2013): the dominance (Bruce, 1910; Jones, 1917), overdominance (Shull, 1908; East, 1936), and epistasis (Schnell and Cockerham, 1992; Yu *et al.*, 1997) hypotheses. With the rapid development of genomic tools, the molecular mechanism of heterosis has been further investigated at the ‘omics’ level. Genome-wide comparative transcriptional profiling between hybrids and their parental lines has been studied in many plants, such as *Arabidopsis* (Wang *et al.*, 2006), maize (Swanson-Wagner *et al.*, 2006), rice (Huang *et al.*, 2006*b*; Wei *et al.*, 2009; Song *et al.*, 2010; Zhai *et al.*, 2013), and *Medicago sativa* (Li *et al.*, 2009*b*). These studies have revealed that the differentially expressed genes (DGs) caused by the interaction of two different sets of parental genomes are potentially involved in controlling the superior performance of heterosis (Zhang *et al.*, 2013). In addition, genomic dosage effects (Yao *et al.*, 2013), allelic variations (Springer and Stupar, 2007; Schnable and Springer, 2013), and epigenetic modifications (He *et al.*, 2013) also contribute to the molecular mechanism of heterosis.

Non-coding small RNAs (sRNAs) in plants, mainly including the microRNAs (miRNAs) (Bartel, 2004) and small interfering RNAs (siRNAs) (Hamilton and Baulcombe, 1999), play important roles in development, stress response (Mallory and Vaucheret, 2006), and heterosis (Baranwal *et al.*, 2012; Ng *et al.*, 2012; Chen, 2013) by inhibiting gene expression. Ha *et al.* (2009) first reported sRNAs involved in the molecular mechanism of heterosis in *Arabidopsis* interspecific hybrids and allopolyploids. Their results suggested that the expression variation of miRNAs, as well as a new class of endogenous siRNAs, *trans-*acting siRNAs (ta-siRNAs), derived from the *TAS* loci via the miRNA-dependent biogenesis pathway (Allen *et al.*, 2005; Liu *et al.*, 2007), led to changes in target gene expression and might result in the phenotypic variation in hybrids. He *et al.* (2010) and Chen *et al.* (2010) compared the expression profiles of sRNAs in seedlings between two rice subspecies (*japonica* cv. *Nipponbare* and *indica* cv. *93-11*) and their reciprocal hybrids by high-throughput sequencing and microarray technology, respectively, and found differential expression of miRNAs between hybrids and their parents. The miRNA transcriptomes were also studied in cultivated and wild tomato seedlings and their hybrids (Shivaprasad *et al.*, 2012), as well as in embryos of a maize hybrid and its parental inbred lines (Ding *et al.*, 2012). However, the interaction between miRNAs and their potential target genes in heterosis of hybrid rice is poorly described.

Previously, we investigated the transcriptional and physiological metabolism changes between super-hybrid rice and its parents (Wei *et al.*, 2009; Song *et al.*, 2010) and found that DGs were significantly enriched in photosynthesis and carbon-fixation pathways, and most of the key genes in the carbon-fixation pathway were upregulated in F_1_ hybrid rice. DGs were mapped to the yield-related quantitative trait loci (QTL), and were involved in the circadian-rhythms and light-signalling regulatory network, suggesting a relationship between DGs and phenotypic changes in hybrid rice. In this study, to determine the roles of sRNAs and their interaction with their target genes in heterosis of hybrid rice, we performed transcriptomic analysis of sRNA and mRNA of flag leaves and panicles of the F_1_ super-hybrid rice *LYP9* and its parents at the grain-filling stage, using next-generation sequencing technology.

## Materials and methods

### Plant materials

Super-hybrid rice *Liang-You-Pei-Jiu* (*LYP9*) and its parental lines, the sterile line *Pei-Ai64s* (*PA64s*) and the restorer line *93-11* (Lu and Zou, 2000; Yu *et al.*, 2002), were planted in a rice field under the same environmental conditions. Each sample had at least three biological replications to minimize systematic errors, with around six to eight flag leaves and panicles per plant, and were harvested at the grain-filling stage. All the plant tissues were pooled in each sample, frozen in liquid nitrogen and stored at –80 °C for total RNA extraction.

### sRNA library construction and sequencing

Total RNAs were extracted from the collected flag leaf and panicle samples at the grain-filling stage of the hybrid rice and its parents using TRIzol Reagent (Invitrogen) according to the manufacturer’s protocol. sRNAs of 18–30 nt were isolated from total RNA by polyacrylamide gel electrophoresis, and ligated to sequencing adapters for reverse transcription (RT)-PCR amplification to generate sRNA libraries as described previously (Hafner *et al.*, 2008). sRNA libraries were subjected to Illumina deep sequencing at Beijing Genomics Institute (Shenzhen, China).

### Sequencing data processing and analysis

After removing the low quality reads and reads <18 nt, and trimming adaptor sequences, clean sequencing reads from sRNA libraries were summarized for length distribution and common/specific sequences between samples, and aligned with the *Oryza sativa* L. ssp. *indica* cv. *93-11* genome (Yu *et al.*, 2002) using the short oligonucleotide analysis package (SOAP) release 2.20 (Li *et al.*, 2009*a*).

### Annotation and classification of sRNAs

sRNA clean reads were aligned with non-coding RNAs [ncRNAs, including rRNAs, tRNAs, small nuclear RNAs (snRNAs) and small nucleolar RNAs (snoRNAs)] in NCBI GenBank release 189 (ftp://ftp.ncbi.nih.gov/genbank/) and Rfam release 10.1 (http://rfam.sanger.ac.uk/); rice miRNA precursors in miRBase release 19 (http://www.mirbase.org/); rice *trans-acting siRNA 3* (*TAS3*) genes (Zhu *et al.*, 2008), rice natural antisense transcripts (NATs) in PlantNATsDB release 1.3 (Chen *et al.*, 2012), repeat sequences extracted from rice genome by RepeatMasker release 3.2.9 (http://www.repeatmasker.org/); and gene transcripts in Rice Genome Annotation release 6.1 (Ouyang *et al.*, 2007), for ncRNA, miRNA, ta-siRNA, natural antisense transcript-generated siRNA (nat-siRNA), repeat-associated siRNA (ra-siRNA), and protein-coding gene annotation, respectively. sRNA annotation followed a priority rule for classification to avoid redundancy: ncRNA (in which GenBank > Rfam) > known miRNA > ta-siRNA > *cis*-nat-siRNA > *trans*-nat-siRNA > ra-siRNA > exon > intron.

### Expression profiling of known miRNAs and ta-siRNAs

The distinct clean reads of sRNAs that perfectly matched the mature sequences of known rice miRNAs (miRBase release 19) and ta-siARFs (Zhu *et al.*, 2008) were used to estimate the expression level of each rice miRNA and ta-siRNA. The initial counts of clean reads from different samples were normalized into reads per million mapped reads (RPM) for expression amounts. Differentially expressed sRNAs (DES) were analysed by comparing the expression amounts between hybrid and the mid-parental value (MPV), using the edgeR package (Robinson *et al.*, 2010) version 3.5.27. The resultant *P* values were adjusted for false discovery rate (FDR) and only adjusted *P* values of ≤0.01 were considered statistically significant. The hierarchical clustering tree of DES in different libraries was generated by MultiExperiment View (Saeed *et al.*, 2006) version 4.9 with the average linkage method.

### RNA sequencing (RNA-Seq) analysis

The total RNAs were extracted as described above, with the same materials as used in the sRNA sequencing. The oligo(dT)-enriched mRNA was fragmented into short fragments of approximately 200 nt for cDNA synthesis and sequencing adapters ligation. The libraries were subjected to Illumina deep sequencing at Beijing Genomics Institute. After quality control, raw reads of RNA-Seq were filtered into clean reads and aligned with the gene models in the Rice Genome Annotation release 6.1 with SOAP release 2.20 (Li *et al.*, 2009*a*). The gene expression level was calculated by using reads per kb of transcriptome per million mapped reads (RPKM) method (Mortazavi *et al.*, 2008), with unique position matched reads. DGs were defined by comparing the expression levels between hybrid and MPV, using the edgeR package (Robinson *et al.*, 2010) version 3.5.27, with a significance threshold of FDR-adjusted *P* values of ≤0.01. Functional category analysis of DGs was performed using the Kyoto Encyclopedia of Genes and Genomes (KEGG) orthology-based annotation system (KOBAS) version 2.0 (Xie *et al.*, 2011), using the NCBI Entrez gene ID of DGs as input.

### Prediction and functional analysis of target genes

Target genes of DES were predicted from the gene transcripts in the Rice Genome Annotation release 6.1 (Ouyang *et al.*, 2007) by the plant sRNA analysis toolbox psRobot (Wu *et al.*, 2012) with the additional threshold of target site conservation and degradome data supported. Correlations between the expression level of DES and corresponding DG targets were analysed via the Pearson correlation coefficient method (Rodgers and Nicewander, 1988). Transcription factor (TF) annotations and classifications of DES targets were according to the PlantTFDB release 2.0 (Zhang *et al.*, 2011). Gene Ontology (GO) enrichment analysis of DES targets was performed using the rice oligonucleotide array database (ROAD) web tools (Cao *et al.*, 2012) with a significance threshold of *P*≤0.01 calculated by the hypergeometric statistical test method.

### Real-time quantitative RT-PCR (qRT-PCR) analysis

Total RNA was extracted from flag leaves at the grain-filling stage of the *LYP9* hybrid rice combination, and used as template for reverse transcription with miRNA-specific stem–loop RT primers (Chen *et al.*, 2005) or gene-specific RT primers (Supplementary Table S12 at *JXB* online) to synthesize the first-strand cDNAs, which were used for SYBR Green (Invitrogen)-monitored qRT-PCR performed on an ABI PRISM 7900HT Fast Real-Time System (Applied Biosystems). The experiment was performed with three biological replicates, using U6 snRNA and *ACT1* as internal references for qRT-PCR of DES and target genes, respectively. The relative expression values of DES or genes in different samples were calculated by the 2^–ΔΔ*C*T^ method (Livak and Schmittgen, 2001).

### Regulatory network analysis

Pathway Studio software (Nikitin *et al.*, 2003) version 9.0 (Ariadne Genomics, Elsevier) was applied to build the regulatory network of DES and their target genes with the NCBI Entrez gene ID (Maglott *et al.*, 2011) as input, via global literature analysis by searching the direct interactions in the ResNet Plant Database version 4.0 release 2012H2.

### Mapping the rice genes to QTL

Rice QTL data, including physical positions on the MSU rice genome release 6 and classification of trait category, were obtained from the Gramene database (Youens-Clark *et al.*, 2011). Coordinates of rice *MIR* and *TAS3* genes were acquired by alignment with the MSU rice genome release 6 (Ouyang *et al.*, 2007) using the BLAST-Like Alignment Tool (BLAT) (Kent, 2002). Based on the genomic positions of both gene loci and QTL, rice genes were mapped to the QTL. QTL of small intervals of <100 genes were extracted and subjected to co-localization with genes in the rice chromosomes. *P* values in QTL trait enrichment analysis of DES and target genes in QTL of small intervals were calculated by the hypergeometric test of the GeneProf webtool (Halbritter *et al.*, 2012), using total gene loci including *MIR* and *TAS3* loci in rice genome as reference.

### Accession numbers

The high-throughput sequence data reported in this paper has been deposited in GEO with accession number GSE51468.

## Results

### sRNA sequencing and data processing

Flag leaves and panicles at the grain-filling stage play important roles in rice yield. To dissect the role of sRNAs in hybrid vigour, we constructed sRNA sequencing libraries of flag leaves and panicles of the super-hybrid rice *LYP9* combination at the grain-filling stage. The above hybrid rice combination includes F_1_ hybrid *LYP9* and its parental lines including the male-sterile line *PA64s* and the restorer line *93-11*. After sequencing, we obtained 63 356 633 high-quality 18–30 nt sRNA clean reads, in which 10 963 733 (*PA64s*), 12 717 274 (*93-11*), and 11 462 878 (*LYP9*) reads were from flag leaves, and 8 474 866 (*PA64s*), 9 147 347 (*93-11*), and 10 590 535 (*LYP9*) reads were from the panicles, respectively; these corresponded to 2 982 601, 3 652 768, 3 214 069, 3 407 271, 3 534 763, and 3 854 243 unique reads in each library (Supplementary Table S1 and Fig. S1 at *JXB* online). Of the sRNA reads in these libraries, 74.1–89.1% of total reads, corresponding to 48.2–81.4% unique reads, matched the *indica* rice *93-11* genome perfectly (Supplementary Table S1).

**Fig. 1.**
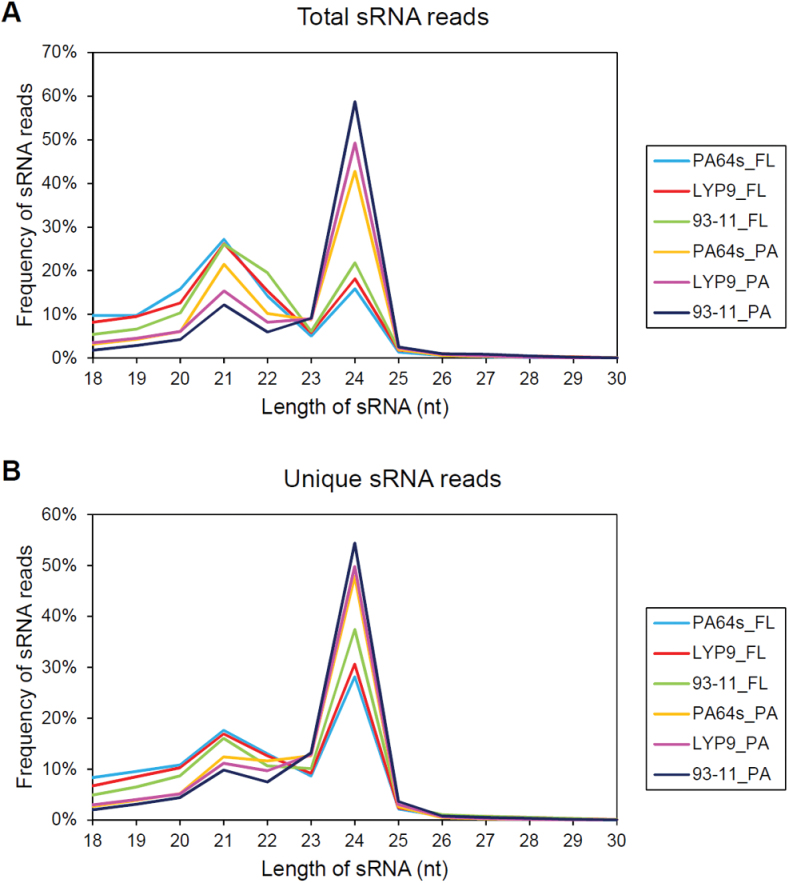
Length distribution of sRNAs. (A) Total sRNAs in different libraries. (B) Unique sRNAs in different libraries. FL, flag leaves. PA, panicles.

In these libraries, 84.6% on average of the total sRNAs were 20–24 nt (a typical size of Dicer-derived sRNAs; Henderson *et al.*, 2006), and of these the 21 and 24 nt sRNAs formed the majority. In the libraries of flag leaves, numbers of 21 nt sRNAs slightly exceeded those of 24 nt sRNAs, whereas in panicles, 24 nt sRNAs were much more numerous than 21 nt sRNAs (Fig. 1A). Of the unique reads, the 24 nt sRNAs were most abundant in all six libraries (Fig. 1B).

To annotate and classify the total sRNAs, the sRNA clean reads were first aligned with the known ncRNAs, including rRNAs, tRNAs, snRNAs, and snoRNAs, in the GenBank and Rfam databases (Supplementary Table S1). Then the remaining sRNAs reads were aligned with the known miRNA precursors of rice in miRBase for miRNA annotation. The results showed that 1 859 743, 1 876 542, and 2 654 408 miRNA reads were detected in flag leaves of *PA64s*, *LYP9*, and *93-11*, respectively, with much lower corresponding numbers of 844 637, 682 363, and 452 645 in panicles (Supplementary Table S1).

The remaining sRNA reads were in turn aligned with the rice *TAS3* genes, NATs, and the repeat sequences in the rice genome, for ta-siRNA, nat-siRNA, and ra-siRNA annotations, respectively (Supplementary Table S1), for the classifications of the three types of endogenous siRNAs with different biogenesis and functions (Vazquez *et al.*, 2010). There were more ta-siRNAs in panicles than in flag leaves, and the four rice *TAS3* genes showed differential expression with each other in the different tissues. In flag leaves, *TAS3a1* had the highest expression level followed by *TAS3b1*, with *TAS3a2* and *TAS3b2* showing very low expression. The order of expression levels in panicles from high to low was *TAS3b1*, *TAS3b2*, *TAS3a1*, and *TAS3a2* (Supplementary Table S1). The ra-siRNAs were the second most abundant sRNAs in flag leaves after miRNAs, and were the most abundant sRNAs in panicles – they were followed in both tissues by nat-siRNAs, of which most were *trans*-nat-siRNAs (Supplementary Table S1).

### Expression profiling of known miRNAs and ta-siARFs in hybrid rice *LYP9* and its parents

We focused on the two kinds of sRNAs, miRNAs and ta-siRNAs (ta-siARFs, the functional ta-siRNAs), which usually direct the mRNA cleavage of target protein-coding genes (Mallory and Bouche, 2008).

Through mapping to the 547 distinct mature miRNA sequences of the 591 known rice miRNA genes in miRBase version 19 and five distinct mature ta-siARF sequences of the four *TAS3* genes (Zhu *et al.*, 2008), a total of 355 sRNAs comprising 350 miRNAs and five ta-siARFs were detected in either flag leaves or panicles. Of these, 244 sRNAs, including 241 miRNAs and three ta-siARFs, were detected in both tissues (Supplementary Table S2 and Fig. S2A at *JXB* online). Based on the identified significance (Robinson *et al.*, 2010) of the expressed sRNAs, 45 and 47 DES between hybrid and MPV were identified in flag leaves and panicles, respectively (Supplementary Tables S3 and S4). This yielded in total 69 DES, comprising 67 miRNAs and two ta-siARFs, in both tissues. This result indicated the obvious expression changes of sRNAs between the F_1_ hybrid and its parents. Among them, 23 DES were detected in both tissues, but 22 of 45 (48.9%) unique DES in flag leaves and 24 of 47 (51.1%) in panicles, respectively, which exhibited the tissue-specificity of DES (Supplementary Fig. S2B).

**Fig. 2.**
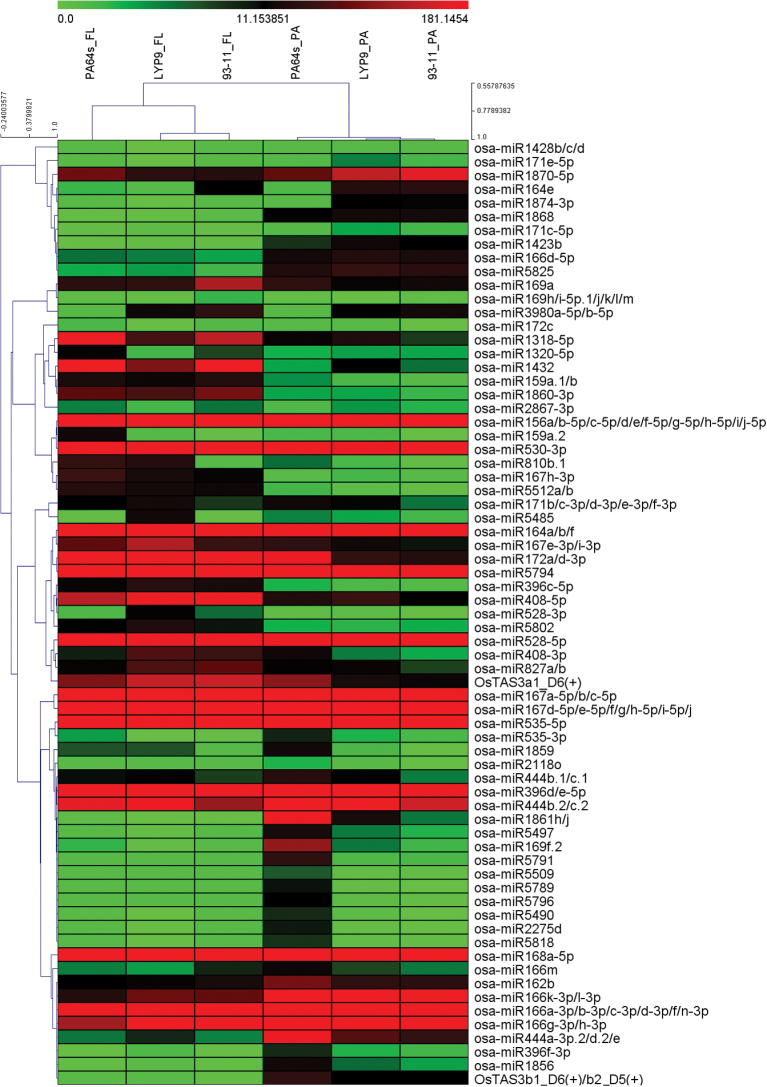
Hierarchical cluster analysis of DES. The hierarchical clustering tree of 69 DES in different libraries of flag leaves (FL) and panicles (PA) was generated by MultiExperiment View version 4.9 using the average linkage clustering method. Read abundance (RPM) is denoted by colour; red and green represent high and low expression levels, respectively.

**Fig. 3.**
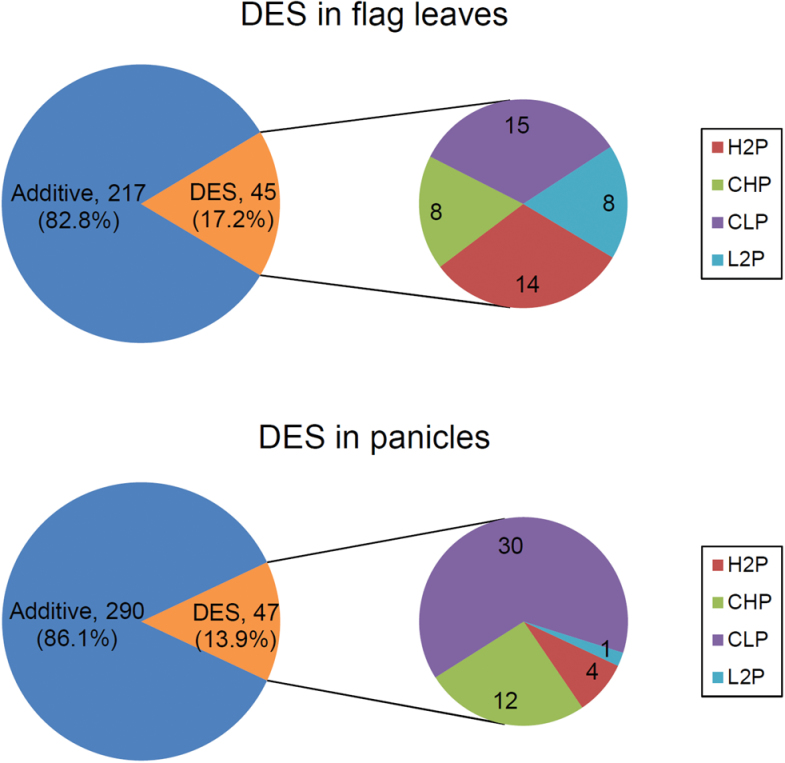
Expression patterns of DES in flag leaves and panicles. Additive represents additively expressed sRNAs without significant differential expression.

Hierarchical cluster analysis was used to investigate the similarity of DES expression profiles, and showed that libraries from different cultivars of the same tissue formed the primary groups. The DES profiles of *LYP9* were much closer to paternal line *93-11* than to maternal line *PA64s*, both in flag leaves and in panicles at the grain-filling stage. This was consistent with our previous study (Wei *et al.*, 2009) on the transcriptional profiles of the *LYP9* hybrid combination (Fig. 2). Comparison of DES expression levels between the F_1_ hybrid and its parents enabled classification of the DES into four patterns: higher than both parents (H2P), close to higher parent (CHP), close to lower parent (CLP), and lower than both parents (L2P) (Fig. 3). Similar to our previous result for transcriptional profiling of the *LYP9* hybrid rice combination (Wei *et al.*, 2009), the CHP and CLP expression patterns of DES in panicles accounted for the majority of all DES (89.4%, Fig. 3).

### RNA-Seq analysis of super-hybrid rice *LYP9* and its parental lines

To correlate DES with their target genes, we also compiled transcript profiles of flag leaves and panicles of *LYP9* hybrid rice and its parental lines (the same materials used in the sRNA analysis) by RNA-Seq. Mapping the RNA-Seq reads to the gene models in Rice Genome Annotation release 6.1 (Ouyang *et al.*, 2007) gave 11 107 734, 11 526 473, and 11 709 849 unique-matched reads of *PA64s*, *LYP9*, and *93-11* from flag leaves, respectively; and corresponding values of 11 448 322, 11 949 274 and 12 333 163 from panicles (Supplementary Table S5 at *JXB* online). Of the detected 40 754 transcripts either in flag leaves or in panicles of the *LYP9* hybrid rice combination (Supplementary Fig. S2C), 7782 (23.2%) and 7629 (19.7%) were differentially expressed between the hybrid and MPV in flag leaves and panicles, respectively (Supplementary Tables S6 and S7 at *JXB* online). Of these, 3229 transcripts were differentially expressed in both tissues (Supplementary Fig. S2D). Among the four expression patterns of DGs, the CHP and CLP patterns were dominant in panicles (69.1%, Supplementary Fig. S3B at *JXB* online), which was similar to the DES patterns.

We employed KEGG annotations to reveal the potential functions of DGs in the hybrid rice *LYP9* combination. Functional category analysis by KOBAS (Xie *et al.*, 2011) showed that DGs were involved in 18 functional categories including two of the mostly enriched categories – carbohydrate metabolism and energy metabolism (Supplementary Fig. S3C), which was consistent with our previous result (Wei *et al.*, 2009).

### Correlation analysis between DES and their potential target genes

**Fig. 4.**
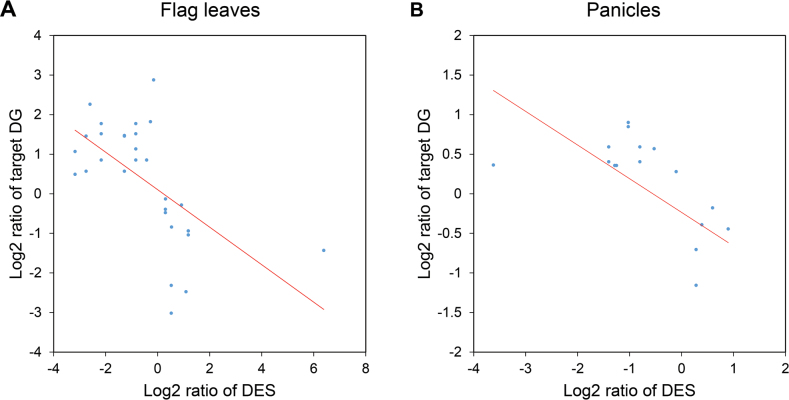
Expression correlation analysis between DES and target DGs in flag leaves (A) and in panicles (B). The Pearson correlation coefficient method (Rodgers and Nicewander, 1988) was used in the correlation analysis.

The target genes of DES were predicted from the gene models in Rice Genome Annotation release 6.1 (Ouyang *et al.*, 2007) via psRobot (Wu *et al.*, 2012) analysis. The result indicated that 34 of 69 DES had recognized target genes (Table 1), and a total of 176 target gene transcripts were predicted (Supplementary Tables S8 and S9 at *JXB* online), among which 123 (69.9%) transcripts were common to both tissues (Table 1). We further investigated the correlations between DES and DG targets by the Pearson correlation coefficient method (Rodgers and Nicewander, 1988). There were significant negative correlations between the expression levels of 23 (67.6%) DES either in flag leaves or in panicles and corresponding target DGs (Fig. 4 and Supplementary Table S9).

**Table 1.** Annotation of DES target genes

Sample name	DES^*a*^	Target transcripts	Target loci	TF transcripts (%)^*b*^	TF^*c*^	TF family
Total^*d*^	34	176	110	112 (63.6)	94	13
Flag leaves	25	145	94	102 (70.3)	86	13
Panicles	25	154	92	103 (66.9)	86	11
Common part^*e*^	16	123	76	93 (69.6)	78	11

^*a*^ DES with predicted target genes by psRobot analysis (Wu *et al.*, 2012).

^*b*^ TF annotations of DES targets were according to PlantTFDB (Zhang *et al.*, 2011). Results in parentheses show the percentage of TF transcripts in total predicted DES target transcripts.

^*c*^ TFs encoded by the DES target transcripts according to PlantTFDB.

^*d*^ Union of flag leaves and panicles.

^*e*^ Intersection of flag leaves and panicles

**Fig. 5.**
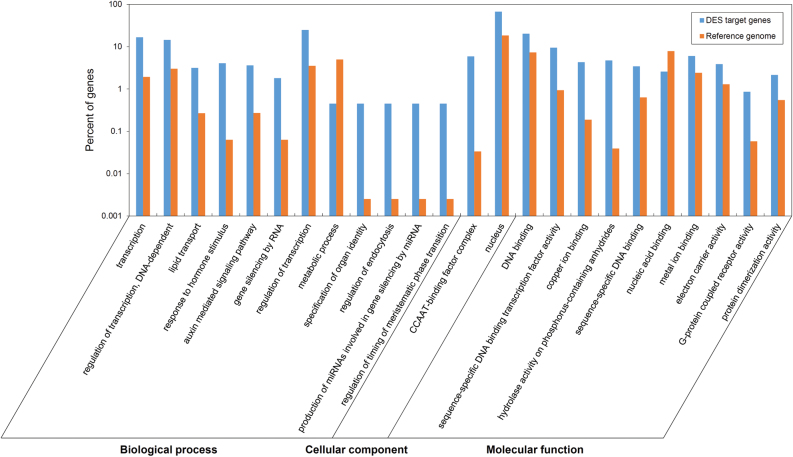
Significantly enriched biological processes, cellular components, and molecular functions identified by GO analysis of DES target genes. The significant GO terms (*P*≤0.01) of target genes were plotted, with the whole genome as the background reference.

According to the plant transcription factor database PlantTFDB (Zhang *et al.*, 2011), the DES targets annotation showed that 112 (63.6%) of the target genes encoded 94 TFs from 13 families (Table 1). GO enrichment analysis by ROAD (Cao *et al.*, 2012) showed that the target genes of DES either in flag leaves or in panicles were significantly enriched in 11 biological processes including the auxin-mediated signalling pathway and regulation of transcription, two cellular components including the nucleus and the CCAAT-binding factor complex, and nine molecular functions including sequence-specific DNA-binding TF activity, compared with total genes in the rice genome (*P*≤0.01, Fig. 5).

### Validation of the DES and target genes expression

**Fig. 6.**
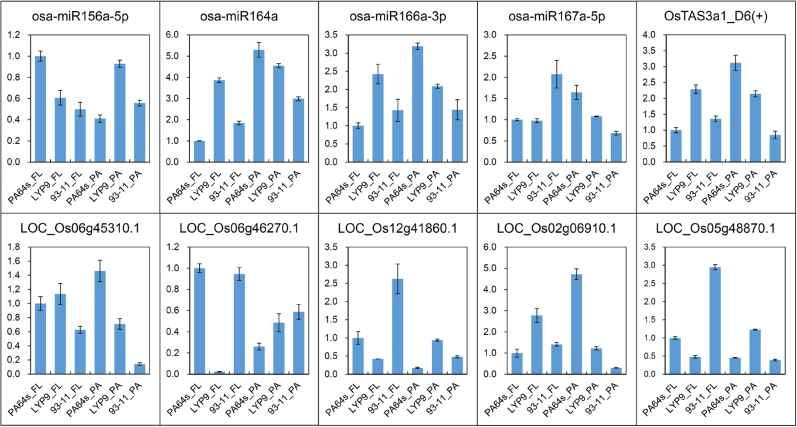
qRT-PCR analysis of DES and target genes. U6 snRNA and *ACT1* were used as internal references for the qRT-PCR of DES mature sequences and target gene transcripts (corresponding vertical lower figure), respectively. The *y*-axis represents the relative gene expression level in different samples. Error bars indicate the standard deviation (SD) for three biological replicates. FL, flag leaves. PA, panicles.

To validate the expression levels of DES identified in the sRNA sequencing and their potential targets, five DES and five of their target genes were randomly selected for examination by real-time qRT-PCR in flag leaves and panicles of the *LYP9* hybrid rice combination. qRT-PCR revealed that all five sRNA mature sequences in flag leaves [osa-miR156a-5p, osa-miR164a, osa-miR166a-3p, osa-miR167a-5p, and OsTAS3a1_D6(+)], as well as three of four DES in panicles (osa-miR156a-5p, osa-miR166a-3p, and osa-miR167a-5p), showed similar expression patterns with the sequencing data (Fig. 6), demonstrating the high quality of sRNA sequencing. Moreover, expression of the potential target genes generally had opposite trends to the corresponding DES (Fig. 6), which confirmed the negative regulation by DES.

### Regulatory network between DES and their target genes

**Fig. 7.**
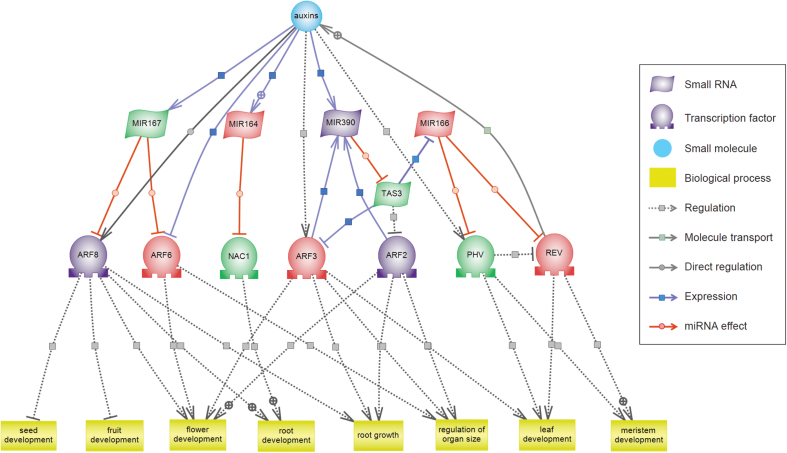
Construction of the auxin signalling regulatory network. DES and their target genes were used for direct interaction analysis by Pathway Studio 9.0. Upregulated, downregulated and unchanged sRNAs and targets in hybrid rice were represented with red, green and purple, respectively. TAS3, *trans*-acting siRNA 3; ARF2, auxin response factor 2; ARF3, auxin response factor 3; ARF6, auxin response factor 6; ARF8, auxin response factor 8; NAC1, NAM/ATAF/CUC 1; PHV, PHAVOLUTA; REV, REVOLUTA.

Genes often interact with other genes to accomplish complex biological processes, as do miRNAs, ta-siRNAs, and their target genes. Using Pathway Studio software (Nikitin *et al.*, 2003), the ‘gold standard’ of pathway analysis, we investigated the roles of the interaction network between DES and their target genes in heterosis of hybrid rice (Fig. 7). The results showed that the regulatory factors of the auxin signalling pathway were regulated by DES. Of these, auxin response factor (ARF) 2 and ARF3 were regulated by ta-siARFs from miR390-directed *TAS3* cleavage, and ARF6 and ARF8 were regulated by miR167. Additionally, pathway analysis showed that the TF NAM/ATAF/CUC 1 (NAC1) was regulated by miR164, and that TFs PHAVOLUTA (PHV) and REVOLUTA (REV) were regulated by miR166. The resulting expression changes of ARFs, NAC1, PHV, and REV facilitated the auxin signalling pathway, which plays important regulatory roles in plant development and growth of important tissues including roots, stems, leaves, flowers, fruit, and seeds (Fig. 7).

### Mapping DES and target genes to QTL in the rice genome

**Fig. 8.**
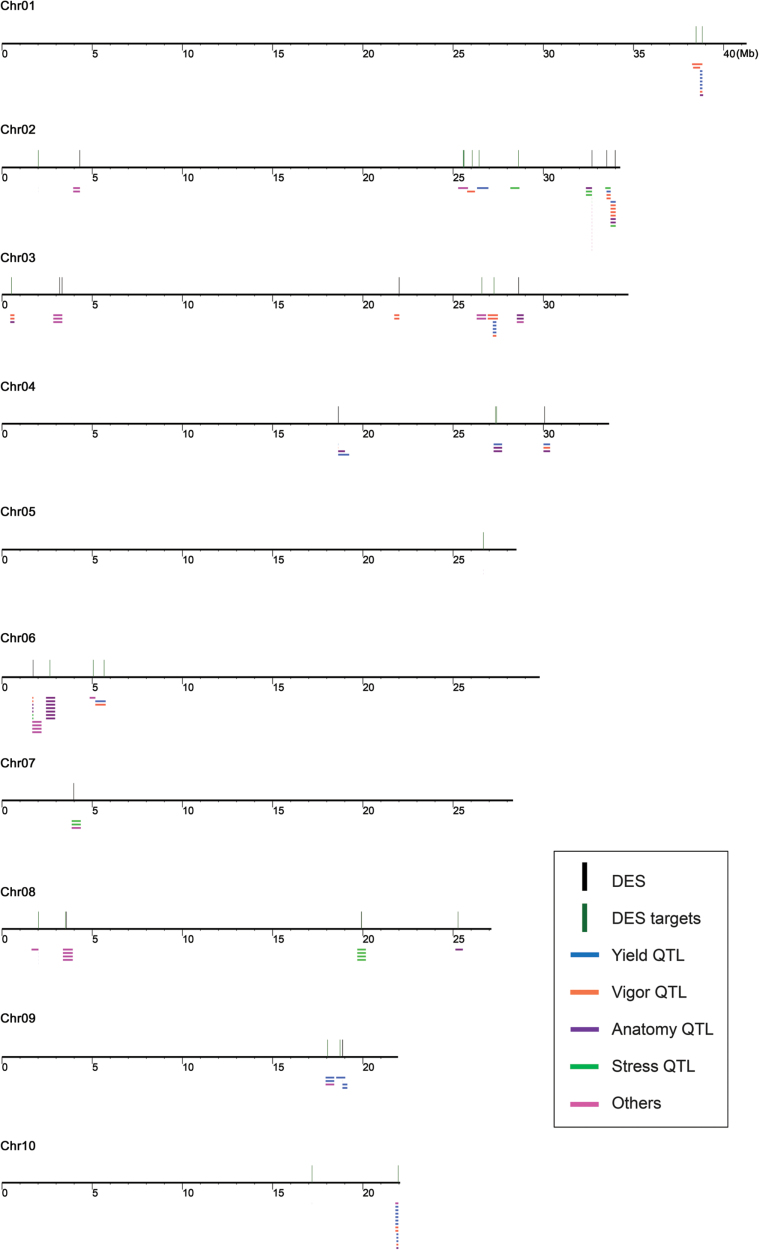
QTL mapping of DES and target genes. The rice QTL from the Gramene QTL database of small intervals (number of genes <100) that harbour DES and the target genes were aligned with the gene coordinates in MSU rice genome release 6. The long horizontal lines represent the rice chromosomes with the scale in Mb, the short horizontal lines represent QTL intervals of different trait categories in different colours, and the short vertical lines represent DES and targets in black and green, respectively. Five classes of QTL traits wee determined: yield, vigour, anatomy, stress (biotic and abiotic stress), and others (biochemical, development, quality, and sterility or fertility).

QTL are identified within genomic interval regions across chromosomes, containing one or more genes associated with the trait being measured. To investigate the association of sRNA expression variation with phenotypic changes in hybrid rice, we mapped the loci of DES and their potential targets to rice QTL in the Gramene database (Youens-Clark *et al.*, 2011), which includes QTL identified for numerous agronomic traits, with genome coordinates. We mapped 211 of 212 (99.5%) loci (comprising 102 DES loci and 110 target loci) to 3214 QTL with 208 traits, which could be classified into nine categories including yield, vigour, and quality (Table 2 and Supplementary Table S10 at *JXB* online). Furthermore, by using all the gene loci in the rice genome as reference, we found 44 (20.8%) DES and target loci significantly enriched in 141 QTL of small intervals spanning less than 100 genes (*P*=0.0008, Supplementary Table S11 at *JXB* online), with 54 traits of nine categories and five classes (Table 2 and Fig. 8). Hybrid rice *LYP9* is advantageous compared with its parents mainly in terms of grain yield, growth vigour, and plant architecture (Lu and Zou, 2000; Yu *et al.*, 2002). As expected, 18 (40.9%), 14 (31.8%), and 16 (36.4%) DES and target loci could be significantly mapped to 38 yield-related, 25 vigour-related, and 26 anatomy-related QTL of small intervals, respectively, among which four DES target loci were mapped to nine yield- or anatomy-related QTL that spanned only one gene (Table 3).

**Table 2.** QTL classification of DES and target genes

Trait category	All QTL in Gramene database			QTL of small intervals^*a*^		
	Trait^*b*^	QTL	Gene^*c*^	Trait^*b*^	QTL	Gene^*c*^
Abiotic stress	44	237	180	9	12	7
Anatomy	43	528	204	10	26	16**
Biochemical	20	95	161	2	2	4
Biotic stress	8	143	169	2	3	2
Development	11	348	206	3	6	5
Quality	34	248	190	3	11	6
Sterility or fertility	8	104	134	2	18	3
Vigour	14	753	208	8	25	14*
Yield	26	758	209	15	38	18*
Total	208	3,214	211	54	141	44**

* and ** denote significant enrichment at *P*≤0.05 and *P*≤0.01, respectively, according to the hypergeometric test in Table S11.

^*a*^ QTL contains gene number ≤100.

^*b*^ Trait classified in Gramene database (Youens-Clark *et al.*, 2011).

^*c*^ Loci number of DES and target genes enriched in the QTL trait.

**Table 3.** DES target genes in yield- or anatomy-related QTL that spanned only one gene

QTL ID^*a*^	Chr^*b*^	Start^*b*^	End^*b*^	Trait category	Trait name	Gene ID^*c*^	Corresponding DES
AQE036	Chr2	2105542	2105840	Yield	Grain yield per panicle	LOC_Os02g04680	osa-miR156/535
AQE049	Chr2	2105542	2105840	Yield	100-Seed weight	LOC_Os02g04680	osa-miR156/535
AQFJ005	Chr8	2114843	2115100	Yield	Grain yield per plant	LOC_Os08g04310	osa-miR528
AQFJ015	Chr8	2114843	2115100	Yield	Panicle tiller ratio	LOC_Os08g04310	osa-miR528
AQGS037	Chr8	2114843	2115100	Yield	Grain yield	LOC_Os08g04310	osa-miR528
AQGS069	Chr8	2114843	2115100	Yield	Grain yield	LOC_Os08g04310	osa-miR528
AQFP005	Chr5	27976107	27977104	Anatomy	Culm thickness	LOC_Os05g48870	OsTAS3-ta-siARF
AQFP027	Chr5	27976107	27977104	Anatomy	Culm thickness	LOC_Os05g48870	OsTAS3-ta-siARF
AQGM008	Chr10	18014265	18014633	Anatomy	Culm length	LOC_Os10g33960	osa-miR166

^*a*^ QTL in the Gramene database (Youens-Clark *et al.*, 2011).

^*b*^ Genomic coordinates of QTL. Chr, Chromosome.

^*c*^ DES target gene loci mapped in the QTL.

## Discussion

### Interaction and functional analysis between DES and their target genes

Recent studies (Ha *et al.*, 2009; He *et al.*, 2010; Groszmann *et al.*, 2011; Barber *et al.*, 2012) have analysed the relationships between sRNA variations and phenotypic changes in types of plant hybrids, providing some information for exploring the role of sRNAs in the heterosis mechanism. However, the role of the interaction between miRNAs/ta-siRNAs and their targets in heterosis is still poorly described in hybrid rice. Previously, we performed a comparative analysis of the transcriptional profiles on the super-hybrid rice *LYP9* combination to approach the regulatory mechanism of heterosis in hybrid rice (Wei *et al.*, 2009). Obvious changes of gene expression were detected in flag leaves and panicles of hybrid rice at the grain-filling stage, which play important roles in grain yield. In this study, we used the same materials to investigate the expression profiles of miRNAs and ta-siRNAs, two class of naturally occurring non-coding sRNAs with clear functions of regulating target mRNAs by causing their cleavage (Chen, 2009), and probed genome-wide for interactions between DES and predicted target genes, and their influence on the vigorous phenotypes of hybrid rice.

Pearson correlation analysis (Rodgers and Nicewander, 1988) indicated that 23 of the 34 (67.6%) DES in two tissues (Supplementary Table S9) displayed opposite expression patterns to the corresponding target DGs (Fig. 4). This result was consistent with the negatively regulating action of plant miRNAs and ta-siRNAs guiding Argonaute 1 (AGO1)- or AGO7-mediated cleavage of the partially complementary target mRNAs (Mallory and Bouche, 2008).

TF annotation analysis indicated that 112 of 176 (63.6%) DES target genes encoded TFs that belonged to 13 families (Table 1), including SBP, NAC, HD-ZIP, ARF, and AP2 (Nakano *et al.*, 2006; Ariel *et al.*, 2007; Guilfoyle and Hagen, 2007; Fang *et al.*, 2008; Guo *et al.*, 2008) (Supplementary Table S9). Intriguingly, all 11 TF families of target genes in panicles were also found in flag leaves (Table 1), suggesting a common transcriptional regulatory mechanism mediated by sRNAs shared in both tissues (Chen, 2009; Jeong *et al.*, 2011).

To further investigate the biological functions of DES target genes, we analysed the GO classifications of the potential target genes of DES by ROAD (Cao *et al.*, 2012) (Fig. 5). The results indicated that the DES target genes were enriched in the biological processes of regulatory functions including regulation of transcription, the response to hormone stimulus, the auxin-mediated signalling pathway, gene silencing by RNA, and regulation of timing of meristematic phase transition, but less in the metabolic process than the reference (Fig. 5) – consistent with the above result that most of the DES targets are TFs.

### DES and their target genes are involved in the auxin signalling regulatory network

To elucidate the potential role of DES and their target genes, we performed interaction network analysis using Pathway Studio, and the result showed that a regulatory network in the auxin signalling pathway was correlated with some of our identified DES and their potential target genes (Fig. 7).

Auxin is a critical factor in plant development that has important influences on the final shape and functions of cells and tissues in all higher plants (Ljung, 2013). In this network, miR164, miR167, and miR390 (Guo *et al.*, 2005; Yang *et al.*, 2006; Yoon *et al.*, 2010), as well as the target genes *ARF6* and *ARF8* of miR167 (Faivre-Rampant *et al.*, 2004; Yang *et al.*, 2006), the target gene *ARF3* of ta-siRNA (Pekker *et al.*, 2005), and the target gene *PHV* of miR166 (Weijers and Jurgens, 2005), could be directly regulated by the plant hormone auxin to execute biological functions in plant development (Ljung, 2013; Pierre-Jerome *et al.*, 2013). *REV* is another target gene of miR166, which is antagonized by *PHV*, and plays a role in altering the auxin polar transport (Zhong and Ye, 1999, 2001; Prigge *et al.*, 2005) (Fig. 7).

The upregulation of miR164 led to a downregulated change in *NAC1*, which transmits auxin signals from the auxin receptor transport inhibitor response 1 (TIR1) to downstream auxin-responsive genes to promote root development (Xie *et al.*, 2000; Guo *et al.*, 2005). NAC1 is a negative regulator of drought tolerance in rice (Fang *et al.*, 2014), and therefore downregulation of *NAC1* might increase the stress resistance of hybrid rice. *ARF6* and *ARF8*, targeted by miR167, have effects on timing of flower maturation, development of seed and fruit, root elongation, and phosphate homeostasis (Goetz *et al.*, 2006; Wang *et al.*, 2014). Downregulation of ta-siARFs generated from miR390-dependent *TAS3* acted on its target genes *ARF2* and *ARF3*, modulating lateral root growth, flowering, leaf senescence and floral organ abscission, and leaf longevity (Ellis *et al.*, 2005; Lim *et al.*, 2010; Marin *et al.*, 2010). The expression changes of miR166 target genes *PHV* and *REV* could direct shoot apical meristem development, lateral organ polarity, and leaf adaxial identity (Huang *et al.*, 2006*a*; Nagasaki *et al.*, 2007; Grigg *et al.*, 2009) (Fig. 7). The downregulation of *TAS3* and resulting upregulation of miR166 might promote the growth of hybrid rice, according to a study of *TAS3* ta-siRNA overexpression transgenic rice with a retarded growth phenotype (Wang *et al.*, 2010). Furthermore, most of the DES in this network were validated, and exhibited opposite trends in expression to the corresponding target genes according to qRT-PCR (Fig. 6). Our results suggest that these DES have a potential role in heterosis in rice, but how exactly the sRNA expression changes contribute to heterosis has yet to be understood.

The auxin signalling network has interactions with the light-signalling network via phytochrome-interacting factors (PIFs) (Hornitschek *et al.*, 2012; Sun *et al.*, 2013). Sentandreu *et al.* (2011) revealed that PIF3 negatively regulated the *Arabidopsis* mitogen-activated protein kinase MPK12, which was proposed to be a negative regulator in auxin signalling (Lee *et al.*, 2009). Our previous study on comparative transcriptional profiling of a hybrid rice combination (Song *et al.*, 2010) revealed significantly differential expression for the rice *PIF3* homologous gene *Os01g18290* (Nakamura *et al.*, 2007). Likewise, *Os01g18290* was also identified as DG in the present study (Supplementary Tables S6 and S7). In *Arabidopsis*, PIF3 interacts with photoreceptors phytochrome A (phyA) and phyB, receiving the light signal at the first step to mediate light signalling (Castillon *et al.*, 2007). The role of PIF3 in rice has not yet been identified completely; it might execute similar biological functions as AtPIF3 (Nakamura *et al.*, 2007). PIF3 regulated the expression of the core circadian-rhythm regulatory gene *late elongated hypocotyl* (*LHY*) by binding to its promoter (Martinez-Garcia *et al.*, 2000), which further facilitates the integration of light-signalling and the circadian-rhythm regulatory network. As a result, PIF3 might play an important role in regulating the downstream genes involved in photosynthesis and carbohydrate metabolic pathways, which will increase the carbon fixation and photosynthetic efficiency in the F_1_ hybrid (Ni *et al.*, 2009; Song *et al.*, 2010; Chen, 2013). *LHY* (*Os08g06110*) and *GIGANTEA* (*GI*) (*Os01g08700*), two important members in the circadian regulatory network, were also identified as DGs in both this study (Supplementary Tables S6 and S7) and a previous study (Song *et al.*, 2010). Shen *et al.* (2012) analysed the genome-wide DNA methylation and transcriptional profiles in *Arabidopsis* and found that the differential expression of the auxin-related genes, including *NAC1* and *vacuolar H* ^*+*^ *-pyrophosphatase 1* (*AVP1*), led to an increase of auxin signalling, which might contribute to the growth vigour of *Arabidopsis* F_1_ hybrids. In the present study, the DES miR164 target gene *NAC1* displayed obvious expression changes and had negatively correlated expression with miR164 (Supplementary Table S9 and Fig. 6), and the *AVP1* homologous gene *Os02g09150* was also identified as a DG (Supplementary Tables S6 and S7). The above results indicated that changes in the complex auxin signalling regulatory network by the interaction between DES and their target genes might contribute to the growth vigour and grain-yield heterosis of hybrid rice.

### DES and their targets were associated with heterosis-related QTL

As an effective tool to investigate the relationship between genotype and phenotype, QTL analysis has been widely used in the crop breeding as well as heterosis studies (Miura *et al.*, 2011; Qu *et al.*, 2012; Fang *et al.*, 2013).

In the present study, we further investigated the correlation of DES and their target genes with heterosis phenotypes using QTL analysis. There were 211 DES and target loci mapped to known rice QTL that were related to nine categories of agronomic traits (Table 2). For example, we mapped the DES gene *osa-MIR156f* and its target gene *Os08g39890*, which encodes SBP-box family protein OsSPL14, to the QTL CQAW25 for tiller number trait in the vigour category (Supplementary Table S10). It has been reported that a point mutation in *OsSPL14* impairs the regulation of OsmiR156 on *OsSPL14*, resulting in ideal rice plant architecture related to tiller number and grain yield, by QTL fine-mapping analysis (Jiao *et al.*, 2010). Moreover, *Os02g04680*, another target gene of miR156, was significantly mapped to the QTL AQE036 and AQE049, which only spanned one gene, for grain yield per panicle and 100-seed weight trait of yield, respectively (Table 3). These confirmed the strong correlations between expression variation of miR156 and yield-related phenotypes in the hybrid rice *LYP9* (Lu and Zou, 2000; Yu *et al.*, 2002).

Furthermore, the anatomy-related QTL of small intervals was the most significant category that DES and their target genes were enriched in (*P*=0.0037, Supplementary Table S11), suggesting the contributions of DES and targets to the plant architecture of hybrid rice. For instance in this category, the ta-siARF target gene *Os05g48870* and miR166 target gene *Os10g33960* could be localized in the QTL AQFP005 for culm thickness trait and AQGM008 for culm length trait that spanned only one locus, respectively (Table 3), implying correlations between DES and the thick culm architecture of the hybrid rice *LYP9* (Lu and Zou, 2000). In addition, the differential expression of miR166 and miR167, which were enriched in the auxin regulatory network in this study, were observed in the hybrid rice *SY63* and its parental lines, *ZS97A* and *MH63*. These two miRNAs were predicted in the QTL intervals of the yield heterotic trait, RG653-G342 and C9098B-RM17 (Fang *et al.*, 2013). These results suggested that the DES-enriched auxin pathway contributed to high-yield traits in different hybrid rice combinations.

In summary, QTL analysis indicated the critical roles of DES and their interactions with target genes in agronomic phenotypic changes of hybrid rice. Our findings provide strong molecular evidence that sRNAs are important regulators that contribute to heterosis in hybrid rice, and open up a new line of deciphering the molecular mechanism of heterosis. Such an understanding of the molecular basis of heterosis can be useful for breeders to take advantage of heterosis in the near future for crop improvement.

## Supplementary data

Supplementary data are available at *JXB* online.

Supplementary Fig. S1. Common and specific sRNA reads in different libraries and tissues of *LYP9* hybrid rice combination.

Supplementary Fig. S2. Common and specific detected miRNAs/ta-siARFs, DES, detected protein-coding genes, and DGs in different tissues.

Supplementary Fig. S3. Expression patterns and functions of DGs in flag leaves and panicles at the grain-filling stage.

Supplementary Table S1. Statistics of annotations and classifications of sRNA reads in different libraries.

Supplementary Table S2. Expression profiling of the known miRNAs and ta-siARFs in different libraries.

Supplementary Table S3. DES in flag leaves of hybrid rice and its parents.

Supplementary Table S4. DES in panicles of hybrid rice and its parents.

Supplementary Table S5. Statistics of RNA-Seq reads in different libraries.

Supplementary Table S6. DG in flag leaves of hybrid rice and its parents.

Supplementary Table S7. DG in panicles of hybrid rice and its parents.

Supplementary Table S8. Predicted target genes of DES with degradome data support.

Supplementary Table S9. List of target genes of DES in flag leaves and panicles.

Supplementary Table S10. List of QTL mapped with DES and target genes.

Supplementary Table S11. Enrichment analysis of DES and target genes in QTL of small intervals.

Supplementary Table S12. Primers used in qRT-PCR.

Supplementary Data

## Abbreviations:

AGO: Argonaute
ARF: auxin response factor
CHP: close to higher parent
CLP: close to lower parent
DES: differentially expressed sRNA
DG: differentially expressed gene
FDR: false discovery rate
GO: Gene Ontology
H2P: higher than two parents
L2P: lower than two parents
miRNA: microRNA;
MPV: mid-parental value
NAT: natural antisense transcripts
nat-siRNA: natural antisense transcripts-generated siRNA
ncRNA: non-coding RNA
PIF: phytochrome interacting factor
qRT-PCR: quantitative reverse transcription-PCR
QTL: quantitative trait loci
ra-siRNA: repeat-associated siRNA
RPKM: reads per kilobase transcriptome per million mapped reads
RPM: reads per million mapped reads
rRNA: ribosomal RNA;
snoRNA: small nucleolar RNA
snRNA: small nuclear RNA
siRNA: small interfering RNA
sRNA: small RNA
ta-siRNA: *trans*-acting siRNA
TF: transcription factor.
