# Supplementary material for: Small RNAs as important regulators for the hybrid vigour of super-hybrid rice
Source: J Exp Bot. 2014 Aug 16;65(20):5989–6002. doi: 10.1093/jxb/eru337 (PMC4203131; doi:10.1093/jxb/eru337)
Supplement: Supplementary Data [file supp_65_20_5989__index.html]

Small RNAs as important regulators for the hybrid vigour of super-hybrid rice — Small RNAs as important regulators for the hybrid vigour of super-hybrid rice — Supplementary Data 

# Small RNAs as important regulators for the hybrid vigour of super-hybrid rice

## Supplementary Data

Data files

**Files in this Data Supplement:**

- Supplementary Data - Supplementary Data
- Supplementary Data - Supplementary Data
