# Supplementary material for: Small RNAs as important regulators for the hybrid vigour of super-hybrid rice
Source: J Exp Bot. 2014 Aug 16;65(20):5989–6002. doi: 10.1093/jxb/eru337 (PMC4203131; doi:10.1093/jxb/eru337)
Supplement: Supplementary Data [file supp_eru337_jexbot124586_file002.pdf]

Small RNAs as important regulators for the hybrid vigor of super-hybrid rice

Lei Zhang, Yonggang Peng, Xiaoli Wei, Yan Dai, Dawei Yuan, Yufei Lu, Yangyang Pan and Zhen Zhu

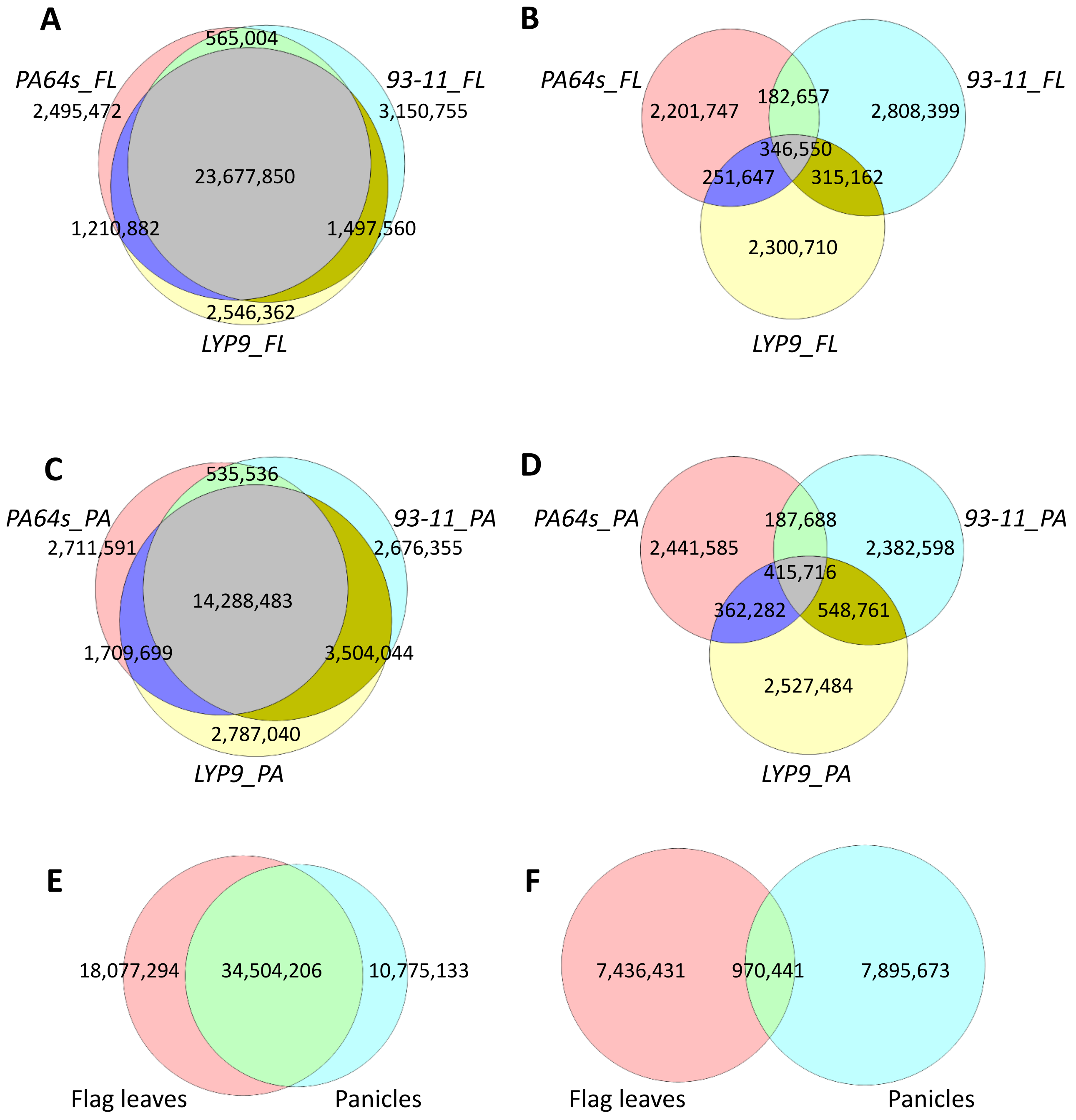

Fig. S1. Common and specific sRNA reads in different libraries and tissues of *LYP9* hybrid rice combination. (A) Venn diagram of total sRNAs in libraries of flag leaves (FL). (B) Venn diagram of unique sRNAs in libraries of flag leaves. (C) Venn diagram of total sRNAs in libraries of panicles (PA). (D) Venn diagram of unique sRNAs in libraries of panicles. (E) Venn diagram of total sRNAs between libraries of flag leaves and panicles. (F) Venn diagram of unique sRNAs between libraries of flag leaves and panicles.

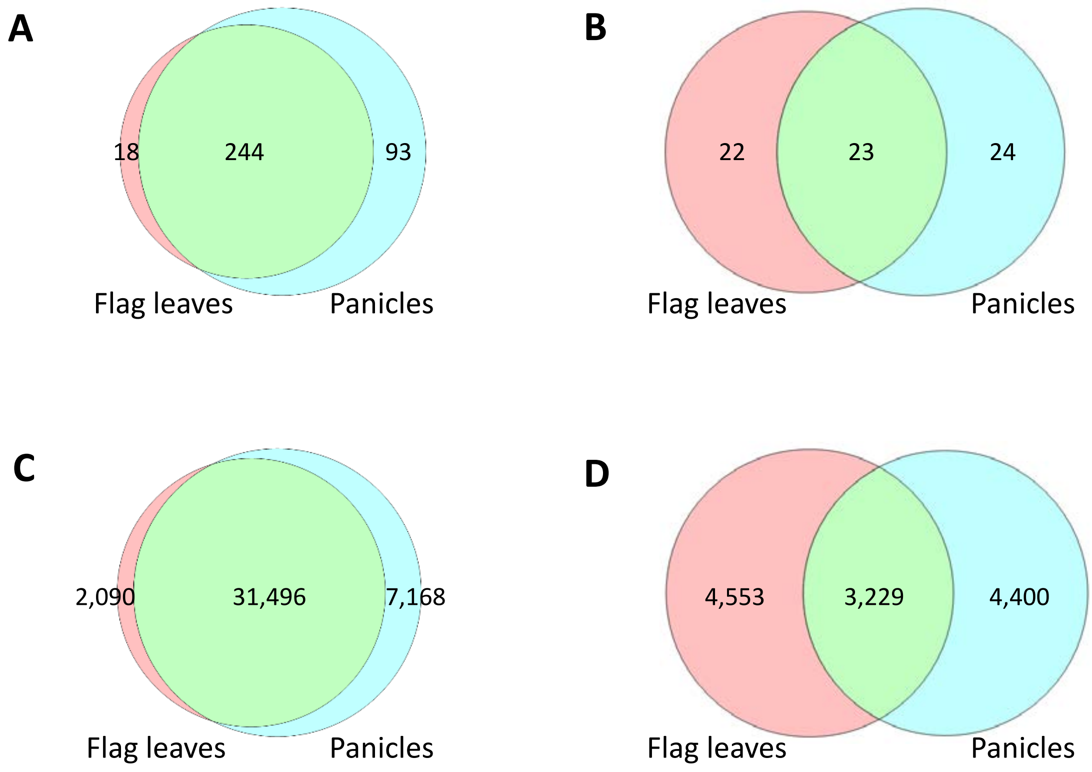

Fig. S2. Common and specific detected miRNAs/ta-siARFs, DES, detected protein-coding genes, and DGs in different tissues. (A) Venn diagram of detected miRNAs/ta-siARFs between flag leaves and panicles. (B) Venn diagram of DES between flag leaves and panicles. (C) Venn diagram of detected protein-coding genes between flag leaves and panicles. (D) Venn diagram of DGs between flag leaves and panicles.

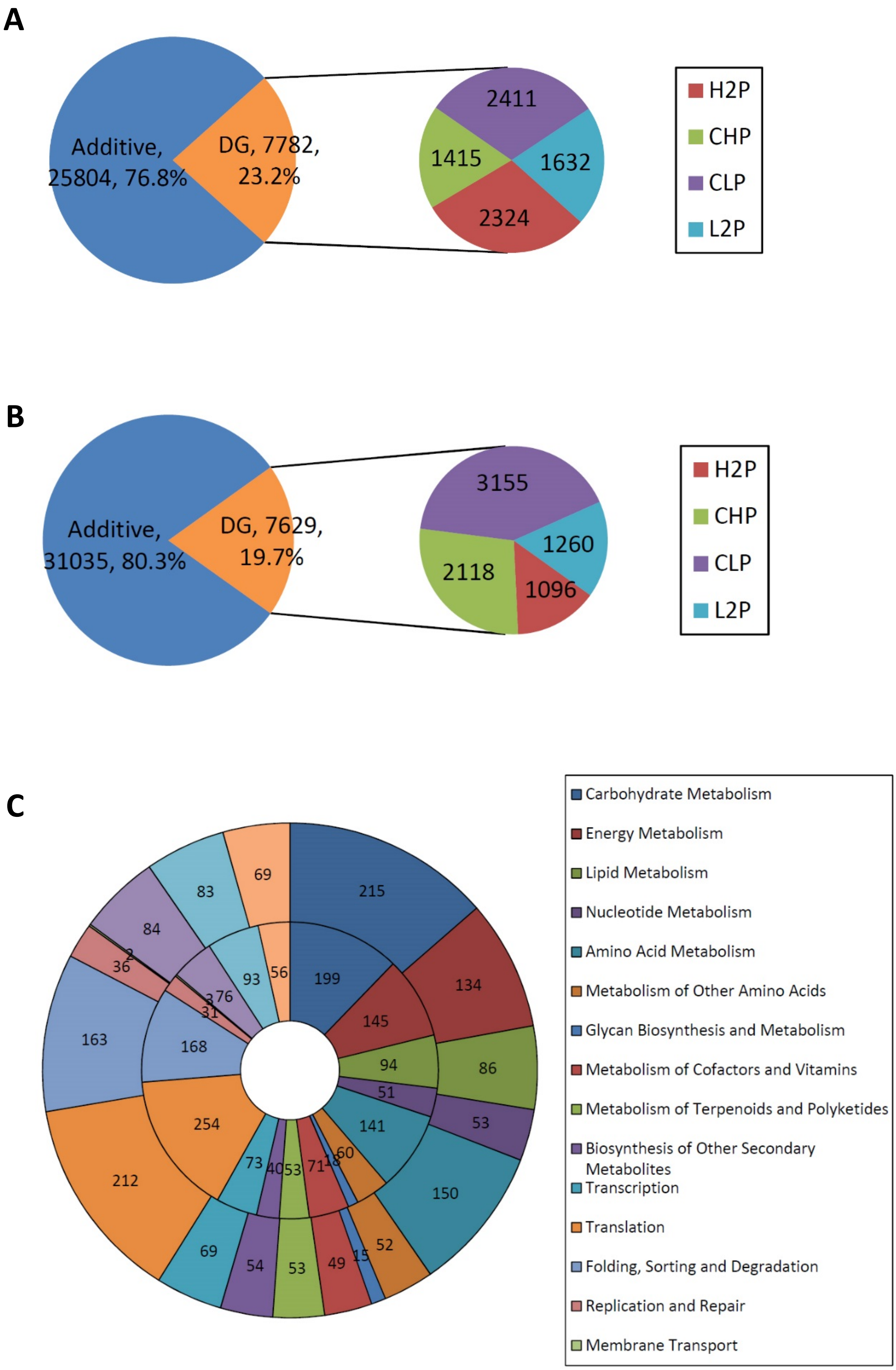

Fig. S3. Expression patterns and functions of DGs in flag leaves and panicles at grain-filling stage. Expression patterns of DGs in (A) flag leaves and (B) panicles. (C) Functional category analysis of DGs by KEGG annotations using KOBAS. Inner circle: flag leaves, outer circle: panicles.
